# Psychological Wellbeing during the COVID-19 Pandemic: The Influence of Personality Traits in the Italian Population

**DOI:** 10.3390/ijerph18115862

**Published:** 2021-05-29

**Authors:** Chiara Rossi, Andrea Bonanomi, Osmano Oasi

**Affiliations:** 1 Department of Psychology, Catholic University of Milan, Largo Agostino Gemelli, 1, 20123 Milano, Italy; chiara.rossi1@unicatt.it; 2 Department of Statistical Science, Catholic University of Milan, Largo Agostino Gemelli, 1, 20123 Milano, Italy; andrea.bonanomi@unicatt.it

**Keywords:** psychological wellbeing, vulnerability, personality traits, COVID-19, coronavirus

## Abstract

Coronavirus disease 19 (COVID-19) has had a strong psychological impact on the Italian population. Italy was heavily affected by the virus before other countries in Europe, experiencing the highest number of deaths. Unknown symptoms in the early stages of the pandemic and the absence of clear transmission links affected people’s wellbeing. Individual personality differences played a key role in perceived psychological wellbeing during the pandemic. The present exploratory study sought to evaluate the impact of COVID-19 on psychological health and identify how psychological wellbeing is influenced by personality traits. A total of 2103 participants (64% female and 36% male) completed an online survey that included the Psychological General Wellbeing Index (PGWBI), the Italian Short Personality Inventory (ITAPI), and a general questionnaire. Descriptive statistics and hierarchical regressions were performed using SPSS 25.0 (IBM Corp., Armonk, NY, USA) (The findings showed poor psychological wellbeing in the Italian population. Young people reported the lowest scores. Vulnerability traits negatively influenced some PGWBI domains, such as the total score (β = −0.62), anxiety (β = −0.55), depression (β = −0.46), positivity and wellbeing (β = −0.51), vitality (β = −0.45), general health (β = −0.12), and self-control (β = −0.52). On the other hand, dynamism traits positively affected vitality (β = 0.12) and positivity and wellbeing (β = 0.14). In other words, personality factors related to vulnerability in particular created risk, whereas dynamism offered protection. The results highlight how COVID-19 helped to trigger anxious and depressive states. People feel helpless and vulnerable when facing new, unexpected conditions caused by the virus. These findings may assist mental healthcare professionals in safeguarding psychological wellbeing during emergencies such as the pandemic.

## 1. Introduction

### 1.1. Background

The coronavirus disease 19 (COVID-19) pandemic has had a strong psychological impact on the Italian population. The non-specific symptoms at the early stages of COVID-19 and the absence of clear transmission links challenged the conventional containment strategies of case isolation and contact quarantine [1]. Several consequences regarding the physical and mental health of individuals, such as fear of infection, lockdown, social isolation, and loneliness, inevitably influenced the wellbeing of the Italian population [2]. In particular, the psychological consequences of the quarantine such as frustration, loneliness, and worries about the future are well-known risk factors for several mental disorders, including anxiety, affective disorders, and psychoses [3,4].

The outbreak of the pandemic represents a unique event in its rapidity of transmission all over the world and does not resemble any other previous traumatic public health event. It became a global health emergency within just a few weeks [5] after originating in China.

The main characteristic of the COVID-19 pandemic is uncertainty; people live without knowing where the virus is, are worried about the future, and are afraid of being infected [6]. To measure these features, Tylor et al. [7] developed and validated the 36-item COVID Stress Scale (CSS) that takes into account the presence of potential traumatic stress symptoms related to COVID-19. Healthcare workers, first and foremost, were seriously affected by the pandemic, as evidenced by several reviews on the topic [8,9,10]. At the same time, the impact of quarantine and physical distancing on the psychological wellbeing and mental health of the general population has been explored in many studies conducted all over the world [11,12,13,14,15].

Qiu et al. [16] discovered that 35% of the population have experienced psychological distress during the epidemic. Sun, L. et al. [17] revealed that 4.6% of the sample experienced a high rate of Post-Traumatic Stress Disorder (PTSD), in particular the female group. Other important studies indicated that, despite the exposure to potentially traumatic events, the development of PTSD is relatively rare (between 5% and 10% of the general population) and could depend on personality traits [18,19].

Personality traits, traditionally conceptualized as dimensions of individual differences, characterize personality structure. These traits could determine states of vulnerability, resilience, or mental distress disorders in individuals. Individual differences in personality traits are one of the factors that might help explain why only some traumatized people develop psychiatric illness [20,21]. Jaksic et al. [22] showed how personality traits influence the development, outcomes, and formation of specific symptoms, such as those of PTSD.

Individual personality differences play a key function in perceived psychological wellbeing and in adopting protective behaviors. Indeed, some recent studies analyzed the role of personality traits on perceived stress during the COVID-19 pandemic [23,24,25,26]. In particular, Nikčević et al. [24] found out that extraversion, agreeableness, conscientiousness, and openness were negatively correlated with generalized anxiety and depressive symptoms. Moreover, neuroticism, health anxiety, and psychological distress caused by COVID-19 were positively correlated with generalized anxiety and depressive symptoms in the American population. A German study [25] noted that extraversion, neuroticism, and openness were among the strongest and most important personality trait predictors of psychological outcomes. Finally, a recent Italian study [26] revealed that negative affect and detachment represented relevant risk factors for reduced emotional wellbeing among the Italian population.

### 1.2. Purpose

At the beginning of the COVID-19 pandemic, the Italian population was heavily affected by the virus before other nations in Europe, and Italy had the highest number of deaths due to coronavirus [27]. From 9 March to 4 May 2020, the life of Italians has been characterized by an increase in COVID-19 cases, a high number of deaths caused by the virus, and social and physical isolation. All these factors, together with fear and uncertainty about the future, could create risk for the onset of psychopathological disorders. In their study, Almeda et al. [28] highlighted increments in both symptomatology and mental disorder incidence caused by the pandemic.

All these findings suggest that personality characteristics are among the key pandemic-related vulnerability factors. Therefore, it is necessary to refer to the growing literature on the importance of considering individual differences in crisis situations.

In line with the previous studies, the current exploratory research has been developed with the aim of (a) evaluating the impact of the COVID-19 pandemic on the psychological wellbeing of the Italian population, and (b) identifying how psychological wellbeing is influenced by personality traits.

**Figure 1:**
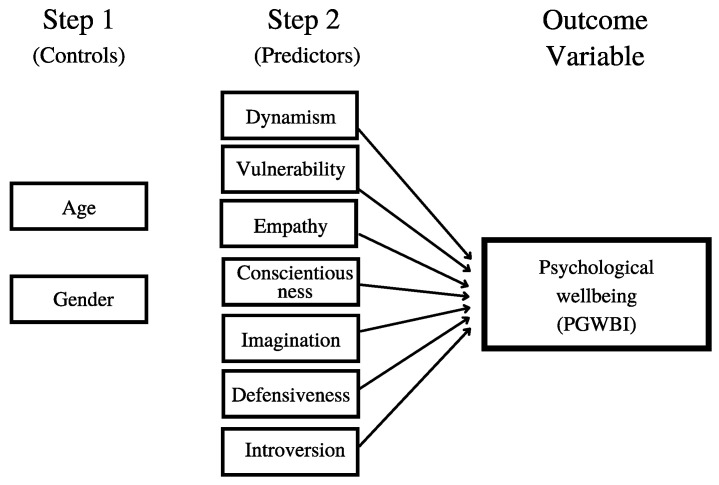
Hypothetical model of the association between personality traits and psychological wellbeing.

Figure 1 represents the theoretical model of this study describing the relationships among all of the used theoretical concepts of the hypotheses.

## 2. Materials and Methods

### 2.1. Participants

The online study was conducted in Italy during the first wave of the pandemic (between 9 March and 4 May 2020). In that period, the number of infections and deaths caused by COVID-19 increased, and lockdown and social isolation were enforced.

Data were collected using an internet-based self-report survey delivered in Italian by *Qualtrics* (Qualtrics, Provo, UT, USA), from 14 to 21 April 2020, at the early stages of the pandemic in Italy. The regression analyses were conducted after calculating the minimum sample size of subjects necessary to estimate the proposed model. We assumed the following criteria: a small effect size (f2 = 0.03), a maximum α value of 0.05, and a recommended test power of 0.80 [29]. By using the software G*Power 3.1.9.7 (G*Power, Düsseldorf, Germany), the minimum sample size required was equal to 264. We obtained an initial sample of 2891 participants. People who answered the questionnaire at a slower pace than average (<10 min) were eliminated.

Participants were recruited through advertisements on social media such as Facebook and Instagram and also word-of-mouth. A social media account was not necessary to complete the questionnaire. IP filtering was applied to guard against duplicate responses to the survey. The online questionnaire required about 15 min to be filled out.

Participants were told the aims, objectives, and procedures of the survey. They were not compensated. Informed consent was requested before starting the online questionnaire. Participation was voluntary, and participants could withdraw from the study at any time. Upon completion of the study, participants were debriefed. Sensitive participant data were anonymized.

In order to administer the questionnaire, ethical approval was obtained on 7 April 2020, by the Ethics Commission for Research in Psychology (CERPS) of the Catholic University of Milan (number: 8–21). The study complies with the guidelines of the 1995 Declaration of Helsinki and its revisions [30].

A total of 2891 people participated in the study. Data collected when a participant had either taken less than 10 min to complete the questionnaire or had not addressed one or more questions were dismissed to ensure the quality of the study. The inclusion criteria were: (a) people over 18 years old, and (b) living in Italy during the COVID-19 outbreak.

For these reasons, the final sample was composed of 2103 people aged 18 to 70 years. The online recruitment produced benefits in terms of feasibility and availability for the participants who were locked at home. In order to assess the influence of COVID-19 on psychological wellbeing, we instructed participants to respond to the questionnaire with reference to COVID-19.

### 2.2. Measures

Participants completed a battery of measures.

(a) Socio-demographics were assessed with a short questionnaire, which included information presented in Table 1.

(b) Psychological wellbeing specific to the current pandemic was assessed with the Psychological General Wellbeing Index (PGWBI) created by Dupuy in 1984 and validated in Italy by Grossi et al. in 2002 [31]. The PGWBI is a 22-item health-related Quality of Life (HRQoL) questionnaire developed in the USA. The test produces a self-perceived evaluation of psychological wellbeing covering the previous four weeks of life. Participants were requested to answer each item considering the impact and implications of COVID-19. The test also identifies six domains understood as levels of wellbeing: general wellbeing, anxiety, depression, positivity and wellbeing, self-control, general health, and vitality. The PGWBI has been validated and used in many countries on large samples of the general population and specific patient groups.

(c) Personality traits were assessed with the Italian Short Personality Inventory (ITAPI-S) developed and validated by Perussia, F. in Italy [32]. The test is based on the Big Five Model and all the guidelines proposed by the American Psychological Association (APA) on mental reactive and personality research [33]. ITAPI-S is the short version of ITAPI-G (including 105 items). This short version includes 28 items measured on a four-point Likert scale from “strongly disagree” to “strongly agree”. The scale measures seven personality traits in line with the Big Five Theory: dynamism and imagination (both considered as openness in the Big Five), defensivity and empathy (viewed as dimensions of agreeableness), vulnerability (represents neuroticism in the Big Five), and finally introversion and consciousness that are the same in both the ITAPI-S and Big Five scales [32,33].

## 3. Data Analysis

Descriptive statistics (i.e., mean, SD, sample size) were performed to present findings for each variable measured. Comparison analyses between the research sample and the normative population of PGWBI Italian validation were conducted. The internal reliabilities of PGWBI and ITAPI-S were analyzed using Cronbach’s α. A correlation analysis between PGWBI’s subscales and ITAPI-S’s subscales were conducted. Finally, to investigate whether personality traits influence the level of psychological wellbeing, a series of hierarchical multiple regression analyses were performed. In each regression, sex and age were firstly included as control variables, then stepwise regression analysis was used to determine how ITAPI-S’s subscales are associated with one of PGWBI’s subscales. Overall, seven regression models were conducted. In the first model, the dependent variable is general health; in the other six models, the dependent variables are each subscale of the PGWBI (anxiety, depression, positivity and wellbeing, self-control, general health, and vitality). To adjust for the 2-sided significance values in a stepwise regression method, the procedure suggested by Moiseev [34] (formula 6) was applied. In particular, to obtain an adjusted *p* = 0.05 for regressions estimating health by personality traits, *p* = 0.006 was used. Statistical analysis was performed using SPSS (IBM Statistical Package for the Social Sciences) version 25.0.

## 4. Results

### 4.1. Descriptive Analyses

There were 2103 valid respondents in total. The sample, aged 18 to 70 years with a mean age of 33.72 (SD = 13.7), was 64% (=1346) female and 36% (=757) male. A total of 1459 people from the North of Italy (69%), 145 from the Center (7%), and 496 from the South and the Islands (24%) answered the questionnaire. Participants’ demographic information is shown in Table 1. The sample had different levels of age and education. Most of them had a high school diploma (42%) or university degrees (36%).

The descriptive statistics and reliability of ITAPI-S are shown in Table 2. The internal reliability of the questionnaire was analyzed using Cronbach’s α.

The ITAPI Cronbach’s α values were not high. In the presence of few elements, the Cronbach’s α coefficient can be lowered to 0.6 [35]. Furthermore, the Cronbach’s α is also not high in the ITAPI-S Italian validation.

The PGWBI was validated in Italy in 2000 on a non-clinical Italian population [31]. A comparison between the average scores and reliability of our research sample and the validation sample of the PGWBI is shown in Table 3 and Table 4.

The asymmetry and kurtosis for the normality of the distribution of our sample were checked. All the PGWBI dimensions are within the acceptable range between −2 and +2 [36].

The reliability measured with Cronbach’s α is similar between the two samples. In the current research, Cronbach’s α was 0.90, showing an excellent internal consistency for the scale. General health and self-control had among the lowest Cronbach’s α values in the series. The authors of the PGWBI [31] validated in Italy had already found low α values for these two domains due to the shortness of the scales. However, general health also correlated with all the other scales of the test, emphasizing the inherently generic nature of its domain. Self-control, on the other hand, was the weakest scale of the questionnaire. This explains why, in our sample, Cronbach’s α of general health (α = 0.43) and self-control (α = 0.66) were low.

For each domain of the PGWBI, high scores correspond to psychological wellbeing. The higher the score, the better the level of wellbeing. High anxiety and depression scores indicate that the population does not experience such negative moods, whereas a low score indicates suffering.

The total score is obtained from the sum of the 22 items of the test and can vary from 0 to 110. The scores have been divided into four areas: severe distress, moderate distress, non-distress, and positive wellbeing.

The total score of the Italian population on which the test was validated was 78, corresponding to the “No-distress” area. The total score of the research sample was 64.5 (SD = 13.1). This score is within the limit of the moderate distress range, slightly above the severe distress range that could cause psychiatric problems. This is an important index of the sample’s psychological health during the first wave of the COVID-19 pandemic.

The perception of anxiety was similar between the two samples. The depression domain was lower in the research sample (m = 8.5; SD = 1.5) than in the PGWBI validation sample (m = 12.4; SD = 2.6). The Italian population may have experienced a depressive state due to the worsening pandemic. The other domains showed similar scores between the two samples, except for Positivity and wellbeing and General Health, which were lower. These results could also be affected by the perception of helplessness and insecurity about the future and the unknown disease.

Both the research sample and the PGWBI validation sample were analysed according to gender. The mean score and the standard deviation split between males and females for each domain are shown in Table 5.

Males of the research sample were more depressed (=8.8) than those of the validation sample of PGWBI (=12.8), as were females (8.3 compared to 12). In the sample of the current study, females in particular reported lower health than men, mainly in terms of anxiety and perception of self-control.

The PGWBI Italian validation authors divided the sample into different age ranges: 18–20; 21–24; 25–29; 30–34; 35–39; 40–44; 45–49; 50–54; 55–64; 65–74; >75.

In the current study, the same division was applied and is shown in Table 6.

Age did not have a crucial effect in the PGWBI validation sample on the perception of psychological health. In the current sample, there were some differences concerning age. Notably, the youngest people (18–24) reported the lowest values across the six dimensions of the PGWBI.

### 4.2. Correlation and Regression Analyses

Pearson’s correlation among the PGWBI and ITAPI-S variables is shown in Table 7.

A series of multiple regressions was conducted considering the PGWBI questionnaire subscales. In each regression, the intercept was one of the PGWBI subscales, and the predictors were the ITAPI-S subscales controlled for gender and age.

For each regression equation, the overall effect size (magnitude of R) was interpreted. Accordingly, in interpreting the results, the greatest emphasis on statistically significant predictors that also had medium or large effects (β > 0.1) was given. The regression analysis of each PGWBI domain is shown in Table 8.

As shown, neither gender nor age affected the results. The vulnerability trait negatively affected the PGWBI total score (β = −0.62), anxiety (β = −0.55), depression (β = −0.46), positivity and wellbeing (β = −0.51), vitality (β = −0.45), general health (β = −0.12) and self-control (β = −0.52). Dynamism as a trait positively influenced vitality (β = 0.12) and positivity and wellbeing (β = 0.14). Despite the differences between genders (Table 5) and ages (Table 6) in the sample, gender and age were not influential predictors of psychological health.

## 5. Discussion

The findings of this study highlighted the poor psychological health of Italians during the COVID-19 outbreak. Young people, in particular, reported low scores on psychological wellbeing tests due to the pandemic. This finding is in line with a recent study [24] and also with a systematic review about adolescence [37]. It is notable considering that COVID-19 has much more serious consequences on the health of older people, who reported no negative psychological health scores.

The findings also showed that the vulnerability trait is the most implicated in the pandemic. Vulnerability has been operationalized by the ITAPI-S authors as being conducive to hardship, fear, and suffering [32].

As we expected, vitality and positivity and wellbeing increased in the presence of dynamism traits and in the absence of vulnerability traits. Dynamism can be considered the same as openness in the Big Five Model, so participants with great creativity, sensibility, and lots of energy (dynamism’s and openness’s sub-dimensions) felt better thanks to these characteristics despite the restriction imposed by the virus. In particular, where total PGWBI score, general health, self-control, vitality, and positivity and wellbeing decreased, there was an increase in the presence of vulnerability traits. These results are largely consistent with predictions because all these domains are always present in the lives of vulnerable people, even during normal times. Furthermore, in subjects with particularly prominent vulnerability traits, there was an increase in the perception of anxiety and depression. Some studies have identified vulnerability as an index associated with neuroticism, known as one of the Big Five personality traits, considered as a risk factor for the onset of anxious and depressive psychopathology [38]. Individuals with high neuroticism scores have a consistent tendency to experience the world as a threatening place. They are quickly distressed, and it is difficult for them to cope with stressful situations [39]. In other words, vulnerable subjects normally perceive negative emotional states, struggle to tolerate environmental stress, and interpret ordinary situations as threatening [40]. The pandemic has exacerbated these experiences and weakened people already considered at risk in normal conditions from a psychological point of view.

The present study highlights the general malaise of the Italian population in terms of psychological health. This is unrelated to age and gender, since these did not appear to be specific predictors of psychological health in the statistical analyses. Psychological distress among the Italian people during the quarantine was also confirmed by another recent Italian study [4].

In Italy, perceptions of psychological wellbeing might have further decreased since 9 March 2020, as the Italian population was heavily affected by the virus before other countries in Europe and was forced to live in social isolation and loneliness. These elements have affected the psychological wellbeing of vulnerable people. The pandemic might have fueled the perception of danger and fear and might have generated anxiety and depression about the unknown.

Similar results have emerged in the Chinese population [41]. In one of the first Chinese studies conducted on approximately 7000 subjects, 35% and 20% of the participants reported generalized anxiety and depression, respectively [42]. In particular, the study showed a high prevalence of general anxiety disorder and poor sleep quality in the Chinese population during the COVID-19 outbreak; female gender was a consistent predictor of this psychological outcome. Regarding age, young adults (aged 18 to 30 years) and the elderly (older than 60 years) showed the highest levels of psychological distress. In this case, young people were at risk with the lowest level of psychological wellbeing, as is the case in the current study.

In line with the previous studies, our findings showed that personality factors related to vulnerability created risk, whereas dynamism offered protection during the pandemic. Vulnerability was defined by Ingram et al. [43] as a stable but unchangeable biological and psychological condition and as an endogenous and latent variable. In particular, stressful events are an environmental risk factor that, when interacting with individual manifestations of vulnerability, could lead to psychopathology. In 2010, the same author [44] proposed a stress–vulnerability–resilience continuum in which the combination of vulnerability–resilience interacts with stressful events to cause the possibility of psychopathological disorders. In this sense, COVID-19 currently represents a potent environmental stressor that interacts with the resilience capacity and with individual vulnerability traits.

## 6. Limitations

Several limitations should be considered in the interpretation of these findings. First, the data were based on self-report questionnaires that could have been influenced by social desirability. Personality traits would be best examined using multiple data sources (e.g., peer reports, experience-sampling measurements, etc.), but it was not possible to incorporate these into a short online survey. Furthermore, some of the measures used for the assessment reported low reliability values. For future research, it is necessary to rethink the use of some questionnaires (already validated) in order to assure the reliability of the instrument. Second, a cross-sectional design was used that allowed significant participation with a broad sample but could not reveal temporal dynamics. Third, participants may not have been representative of the general population. Besides, the prevalence of the female gender and the geographic location such as Northern Italy, the presence of many young people, and also high school diploma were identified as selection bias. Indeed, the sample was mainly composed of a younger population, which also reported lower wellbeing scores. These characteristics may have affected the results. These limitations could be addressed in future studies by ensuring a diverse research design.

## 7. Conclusions

Despite these above limitations, the study demonstrated the need to consider personality traits such as individual differences in crisis situations. In particular, the main finding was that vulnerability traits affected the perception of psychological health during the first wave of COVID-19. The results highlighted that in order to prevent the risk of the onset of episodes of mental disorders, it is necessary to take care not only of those who are already suffering from psychological disorders but also of the non-clinical population. The findings could also be useful for Italian psychologists and family doctors who take care of people suffering from depression, anxiety, or difficulty due to the stressful situation caused by the coronavirus to orient their care in light of what has emerged.

The outbreak of COVID-19 has been and continues to be traumatic for the entire Italian population. In response to the fear and stress of contagion, the majority of mental disorders following COVID-19 may be “reactive” in nature. Some degree of sadness, anxiety, fear, anger, paranoia, short-term adjustment issues, and long-term adaption to the uncertain future is perhaps reasonable or expected [45]. As we demonstrated, these responses vary according to individual differences influenced by personality traits.

COVID-19 triggered anxious and depressive states. Despite all the resources employed to counteract the spread of the virus, additional global strategies are needed to handle the related mental health issues.

The research, therefore, highlights the role that personality plays during crises, especially with respect to the pandemic. As resources could be particularly scarce during this serious pandemic, timely psychological support could take many different forms. In particular, to maximize existing resources, it could be useful to promote good mental health at home, including telemental health services and psychiatric teleconsultation. Social support networks and online support groups could also help people to stay connected during this pandemic [46,47]. Further research should examine the nature of the observed poor psychological health of the Italian population to suggest suitable interventions.

## Figures and Tables

**Table 1:** Descriptive statistics of the sample.

Characteristic	Group	*n* (%)
Gender	Female	1346 (64%)
	Male	757 (36%)
Age		2103 (100%)
M (SD)	33.72 (13.7)	
Min-Max	18–70	
Geographical position	North	1459 (69%)
	Center	145 (7%)
	South	478 (23%)
	Islands	21 (1%)
Education	No diploma	4 (0%)
	Primary school diploma	0 (0%)
	Middle school diploma	132 (6%)
	High school diploma	884 (42%)
	Graduate	764 (36%)
	Postgraduate	319 (15%)

**Table 2:** The descriptive statistics and reliability of ITAPI-S.

ITAPI-S	Mean	SD	Skewness	Kurtosis	Cronbach’s α
Dynamism	11.72	2.20	−0.17	−0.16	0.67
Vulnerability	10.14	2.73	0.03	−0.52	0.74
Empathy	12.46	2.06	−0.41	0.05	0.60
Conscientiousness	11.81	2.32	−0.31	−0.29	0.69
Imagination	12.12	2.44	−0.33	−0.51	0.76
Defensiveness	11.72	2.08	−0.14	−0.35	0.52
Introversion	10.71	2.46	−0.09	−0.29	0.66

**Table 3:** Descriptive statistics of the PGWBI for the research sample and validation sample of the instrument.

	Research Sample (2103)				PGWBI Validation Sample (1129)	
PGWBI	Mean	SD	Skewness	Kurtosis	Mean	SD
Total score	64.5	13.1	−0.235	−0.140	78	-
Anxiety	16.1	4.7	−0.527	0.030	17.3	4.9
Depression	8.5	1.5	−0.658	1691	12.4	2.6
Positivity and wellbeing	9.9	3.2	0.176	−0.124	11.8	4.1
Vitality	12.2	3.2	−0.291	0.004	13.4	4
General Health	7.4	1.8	−0.283	1019	11.1	3.1
Self-control	10.4	2.7	−0.495	0.087	11.8	2.7

**Table 4:** The reliability of the PGWBI for the research sample and validation sample of the instrument.

	Research Sample (2103)	PGWBI Validation Sample (1129)
PGWBI	Cronbach’s α	Cronbach’s α
Total score	0.90	0.94
Anxiety	0.87	0.85
Depression	0.77	0.80
Positivity and wellbeing	0.80	0.80
Vitality	0.76	0.77
General Health	0.43	0.70
Self-control	0.66	0.61

**Table 5:** Comparison between the research sample and PGWBI validation sample by gender.

Male	Research Sample (=757)		PGWBI Validation Sample (=543)	
	Mean	SD	Mean	SD
Anxiety	17.2	4.5	18.1	4.6
Depression	8.8	1.5	12.8	2.3
Positivity and wellbeing	10.5	3.3	12.5	3.8
Vitality	12.7	3.3	14.2	3.5
General Health	7.7	1.7	11.5	2.9
Self-control	11.2	2.6	12.1	2.5
**Female**	**Research Sample (=1346)**		**PGWBI Validation Sample (=586)**	
	**Mean**	**SD**	**Mean**	**SD**
Anxiety	15.3	4.7	16.6	5.2
Depression	8.3	1.5	12	2.8
Positivity and wellbeing	9.7	3.1	11.1	4.1
Vitality	11.9	3.1	11.5	2.8
General Health	7.3	1.8	10.7	3.2
Self-control	9.9	2.7	12.7	4.3

**Table 6:** Descriptive statistics of the sample divided by age.

	Anxiety		Depression		Positivity and Wellbeing		Vitality		General Health		Self-Control	
**Range Age**	**Mean**	**SD**	**Mean**	**SD**	**Mean**	**SD**	**Mean**	**SD**	**Mean**	**SD**	**Mean**	**SD**
18–20 (145)	15.55	4.80	8.06	1.82	9.50	2.92	11.54	3.08	7.85	1.54	9.70	2.78
21–24 (524)	14.64	4.87	8.10	1.56	9.29	2.92	11.51	3.19	7.45	1.86	9.70	2.73
25–29 (547)	16.18	4.58	8.48	1.46	10.13	3.06	12.41	3.06	7.53	1.72	10.28	2.60
30–34 (167)	16.24	4.58	8.64	1.46	10.19	3.11	12.08	2.93	7.32	1.78	10.63	2.45
35–39 (105)	16.87	4.40	8.64	1.42	10.67	3.30	12.49	3.17	7.43	1.64	11.01	2.49
40–44 (105)	16.24	4.51	8.77	1.67	10.05	3.38	12.26	3.78	7.18	1.85	10.89	2.54
45–49 (115)	16.23	4.80	8.56	1.51	9.98	3.89	12.46	3.66	7.40	1.68	10.65	3.00
50–54 (128)	16.92	4.49	8.71	1.24	10.71	3.19	13.15	3.29	6.88	1.79	11.03	2.66
55–64 (212)	17.28	4.16	8.79	1.43	10.53	3.31	12.87	3.08	7.22	1.65	11.21	2.43
65–75 (55)	18.17	4.05	9.11	1.42	11.09	3.28	13.37	3.42	7.37	1.97	11.43	2.66
Total = 2103												

**Table 7:** The correlation matrix between the PGWBI and ITAPI-S variables.

ITAPI-S	Total	Anxiety	Depression	Positivity and Wellbeing	Vitality	General Health	Self-Control
Dynamism	0.199 **	0.086 **	0.124 **	0.259 **	0.212 **	0.051 *	0.150 **
	0.000	0.000	0.000	0.000	0.000	0.018	0.000
Vulnerability	−0.627 **	0.566 **	−0.467 **	−0.526 **	−0.465 **	−0.109 **	−0.546 **
	0.000	0.000	0.000	0.000	0.000	0.000	0.000
Empathy	0.056 *	0.029	0.053 *	0.097 **	0.078 **	−0.115 **	0.058 **
	0.011	0.189	0.015	0.000	0.000	0.000	0.008
Conscientiousness	0.023	0.033	−0.024	0.044 *	0.079 **	−0.037	0.062 **
	0.283	0.133	0.275	0.043	0.000	0.094	0.004
Imagination	−0.014	0.032	−0.005	0.037	0.021	0.010	−0.085 **
	0.527	0.145	0.812	0.089	0.332	0.636	0.000
Defensiveness	−0.073 **	0.069 **	−0.026	−0.087 **	−0.048 *	−0.017	−0.050 *
	0.001	0.002	0.240	0.000	0.028	0.425	0.022
Introversion	−0.025	0.024	−0.026	−0.056 **	−0.049 *	0.092 **	0.001
	0.254	0.269	0.234	0.010	0.025	0.000	0.981

* The correlation is significant at the 0.05 level. ** The correlation is significant at the 0.01 level.

**Table 8:** Regression analysis predicting PGWBI’s scores from ITAPI-S’s domains (controlling for gender and age).

Intercept	Predictors	β	t	*p*-Value	R^2^
**Total score**	Gender	−0.07	−4.20	0.000	0.42
	Age	0.05	3.05	0.002	
	Vulnerability	−0.62	−33.69	0.000	
	Imagination	0.08	4.54	0.000	
	Dynamism	0.07	3.66	0.000	
	Introversion	0.05	3.21	0.001	
	Empathy	0.05	2.88	0.004	
**Anxiety**	Gender	−0.07	−4.07	0.000	0.34
	Age	0.06	3.51	0.000	
	Vulnerability	0.56	−29.49	0.000	
	Imagination	0.08	4.53	0.000	
**Depression**	Gender	−0.08	−3.94	0.000	0.24
	Age	0.08	4.21	0.000	
	Vulnerability	0.46	−22.64	0.000	
	Imagination	0.09	4.41	0.000	
	Defensiveness	0.05	2.78	0.006	
**Positivity and wellbeing**	Gender	−0.03	−1.35	0.179	0.32
	Age	0.03	1.45	0.147	
	Vulnerability	−0.51	−26.20	0.000	
	Dynamism	0.14	7.30	0.000	
	Imagination	0.08	4.23	0.000	
	Empathy	0.07	3.70	0.000	
**Vitality**	Gender	−0.01	−0.77	0.444	0.25
	Age	0.07	3.40	0.001	
	Vulnerability	−0.45	−21.78	0.000	
	Dynamism	0.11	5.10	0.000	
	Imagination	0.08	3.81	0.000	
	Conscientiousness	0.05	2.81	0.005	
**General Health**	Gender	−0.08	−3.46	0.000	0.05
	Age	−0.10	−4.29	0.001	
	Vulnerability	−0.12	−5.33	0.000	
	Introversion	0.09	4.02	0.000	
	Empathy	−0.07	−3.26	0.001	
**Self-Control**	Gender	−0.09	−4.87	0.000	0.33
	Age	0.08	4.18	0.000	
	Vulnerability	−0.52	−27.59	0.000	
	Conscientiousness	0.05	2.85	0.004	
	Empathy	0.07	3.51	0.000	
	Introversion	0.06	3.19	0.001	

## Data Availability

The datasets used and/or analyzed during the current study are available from the corresponding author on reasonable request.

## Abbreviations

PTSD: Post-Traumatic Stress Disorder; PGWBI: Psychological General Wellbeing Index; ITAPI-S: Italian short personality inventory; APA: American Psychological Association.
